# User fee removal for the poor: a qualitative study to explore policies for social health assistance in Iran

**DOI:** 10.1186/s12913-022-07629-8

**Published:** 2022-02-24

**Authors:** Manal Etemadi, Mohammad Hajizadeh

**Affiliations:** 1 Department of Health Services Management, School of Health Management and Information Sciences, Iran University of Medical Sciences, Tehran, Iran; 2 School of Health Administration, Faculty of Health, Dalhousie University, Halifax, Canada

**Keywords:** User fee, Social health assistance, Health services, The poor, Iran

## Abstract

**Introduction:**

Removal of user fee for vulnerable people reduces the financial barriers associated with healthcare payments, which, in turn, improves health outcomes and promotes health equity. This study sought to provide policy strategies to reduce user fee at the point of service delivery for the poor in Iran.

**Methods:**

This is a qualitative study carried out in 2018. The purposive sampling method was applied, and 33 experts with relevant and valuable experiences and maximum variation to obtain representativeness and rich data were interviewed. Trustworthiness criteria were used to assure the quality of the results. The data were analyzed based on thematic analysis using the MAXQDA10 software.

**Results:**

The most important issue regarding financial protection against user fee for the poor in Iran is policy integration and cohesion. Differences in access to financial support for user fee coverage among different groups of the poor have led to inequalities in access and financial protection among the poor. The suggested protection policies against the user fee at the point of service delivery in Iran can be categorized into three main categories: 1) basic health social insurance instruments, 2) free health services to the poor outside of the health insurance system, and 3) complementary insurance mechanisms.

**Conclusion:**

Implementing a cohesive social assistance policy for all disadvantaged groups is needed to address inequalities in financial protection against user fee payment among the poor in Iran. Reducing user fee through mechanisms such as deductible cap, stop-loss, variable user fee and sliding fee scale can improve financial protection and enhance healthcare utilization among the poor. A user fee exemption is not enough to remove barriers to access to service for the poor, as other costs such as transportation expenditures and informal payments also put financial pressure on them. Therefore, financial support for the poor should be designed in a comprehensive protection package to reduce out-of-pocket payments for healthcare services, and indirect costs associated with healthcare utilization.

## Introduction

User fee and benefit package design are essential elements of a universal coverage strategy. User fee is seen as part of the overall financing system, not just a tool to increase revenue or reduce demand. The level of user fee is an indicator of the depth of effective insurance coverage and an essential part of the strategic purchasing function [1]. The effects of user fee on the poor are more controversial than any other methods of healthcare financing. In addition to reducing access to health services and welfare for the poor, this method of financing increases the possibility of refusing to receive services due to uncertainty about costs and the shame of not being able to pay. As user fee is considered a source of income for providers, it does not create incentives to exempt the poor from paying these fees [2].

Decreased adherence to treatments and delay in seeking treatments are among the adverse effects of user fee on poor patients [3, 4]. User fee has less adverse effects on middle- and high-income individuals, but poor families and chronically ill people experience severe health effects of user fee due to the reduction in healthcare utilization [5]. There has been growing interest in designing user fee based on individuals’ income level because low-income people are high-volume users and disproportionately face more economic consequences [6].

It is considered unfair to have the same user fee for low-income and high-income groups because the user fee accounts for a higher percentage of total household income for low-income than their high-income individual counterparts. This is against the equity principle of paying to the healthcare system according to the ability to pay [7]. User fee imposes greater economic consequences on the poor because even the smallest payment can represent a significant share of their household income. Income shock due to illness of family members (including income earners) attributes to a greater impoverishment among the poor [8].

User fee places a significant financial burden on people close to the poverty line and has many consequences for low-income patients, especially concerning the management of chronic diseases. There is ample evidence that user fee reduces the utilization of healthcare at the point of service delivery. Since patients are largely unable to distinguish between essential and non-essential care, user fee reduces the use of preventative services and essential medications. Evidence suggested that increasing user fee for medicine is associated with increased inpatient and emergency services in patients with chronic diseases [9].

As a joint initiative sponsored by the World Health Organization (WHO) and the United Nations Children’s Fund (UNCF) and implemented in some African countries, the Bamako Initiative introduced user fee and community participation in local health financing and local resource management. This initiative failed to address equity, efficiency, and sustainability challenges, and further marginalized the poor. While user fee can be considered a source of additional income, it is not a significant source of income for the health sector. Even if the only purpose of user fee is cost recovery, most studies showed that it does not cover the cost of services [10]. Instead of compensating for costs and preventing overuse of services, user fee has eventually become a means of punishing the poor [11]. User fee results in sustainable inequity and makes health systems function, but keep out the poor [12].

Decreasing user fee for vulnerable people (e.g., the poor) reduces the financial problems associated with healthcare payments, which, in turn, improves health outcomes and promotes health equity. Moving from user fee to pre-payments through taxes and premiums is necessary to fund universal health coverage (UHC). Government funding to subsidize healthcare expenditures for low-income and disadvantaged individuals is an effective policy lever to reduce inequity in access and improve health outcomes [13]. User fee removal increases the demand for services by the poor and reduces the financial barriers for the poor to access services. Meanwhile, it does not lead to inappropriate or unnecessary use of services [14–16].

The poverty rate based on $5.50 a day (2011 Purchasing Power Parity) was 14% in Iran in 2018 [17]. Low household income is associated with a higher probability of catastrophic health expenditures in Iran. The highest rate of catastrophic expenditure is observed in the first and second income deciles in Iran, and lack of complementary insurance is an important contributor to catastrophic expenditure among these households [18]. A recent study has shown that 71.7% of catastrophic health expenditures occurred among the poor in Iran [19]. In the context of the current economic sanctions and associated financial crisis in Iran, the importance of protecting the poor against catastrophic healthcare expenditures has become more evident [20].

According to Article 9 of the Universal Health Insurance Law (UHIL), approved in 1994, the per capita health insurance premiums and the amount of user fee should be set based on the income level of the insured groups and the economic and social situation of the country. However, since enacting this law, co-insurance has been applied at a fixed rate to all insured persons equally. This has imposed significant financial pressure on the poor [21]. The co-insurance rate is 10% for all insured persons for inpatient services and 30% for medicines and outpatient services -- except for medicines for special and incurable patients. There is also a fixed co-payment for general practitioners and midwives in rural areas, which is lower than in urban centers.

Approximately 90% of Iranians, including the poor, are under health insurance coverage in a “Bismarckian system” in Iran [22]. Forty-four governmental, non-governmental and para-governmental organizations in Iran, by law, are required to provide financial support for 27 designated vulnerable groups (e.g., unsupported women and children, the poor disabled, the poor elderly, the poor addicts, the unemployed, the mentally ill, the sick with the chronically ill) to access health services [21]. Nevertheless, due to overlapping tasks and insufficient cooperation between these institutions, there is insufficient financial support for the poor to utilize healthcare services [20]. According to a joint report released by the WHO and the World Bank in 2017, Iran is categorized as a low-performing low/middle-income country with for financial protection [23]. There exist wide variations in user fee assistance across different groups of the poor covered by the health insurance funds (i.e., the Imam Khomeini Relief Committee [IKRC] and the State Welfare Organization [SWO], the Universal Health Insurance Fund, and the Rural Insurance Fund) in Iran. The IKRC’s members have additional support for basic health insurance coverage. The SWO’s members do not have a comprehensive policy for supplementary support except for persons with disabilities. Those insured by the Universal Health Fund and Rural Insurance Fund and not affiliated with the IKRC do not have access to any financial assistance to cover their user fee and out-of-package services [24]. The existing evidence suggests that high out-of-pocket payments (OOP) for healthcare in Iran led the poor rural residents to become more impoverished than their urban counterparts [25, 26].

In addition to the above-mentioned health insurance funds, the Social Work Departments in public hospitals provide further support for this group of patients in Iran. The main task of this department is to review the user fee discount applications of the poor patients and grant financial discounts to these patients using the department budget and contact donors and charities to finance the exempted user fees. Uninsured and the insured poor people who are unable to pay their share of the cost of services can ask social workers in hospitals for financial assistance to reduce their bills. This is a passive method of financial support and does not prevent patients from seeking service due to financial incapacity. A recent study showed that most referrals to the Social Work Departments were patients insured by the IKRC and those without insurance coverage [27]. This suggests that even the user fee of public hospitals is not affordable for those covered by the IKRC.

Designing policies related to reducing user fee among the poor in health systems can help achieve the UHC and Sustainable Development Goals. As the issue of the user fee, especially among the socioeconomically disadvantaged population, is a major issue in Iran, the present study aims to provide policy solutions to reduce OOP for healthcare by the poor at the point of service delivery in Iran.

## Methods

This is a qualitative study carried out in 2018. The study population consisted of policymakers, experts and scholars from the Ministry of Health and Medical Education (MOHME), Ministry of Cooperatives, Labor and Social Welfare (MCLSW), the Plan and Budget Organization (PBO), the Parliament, major health insurance organizations (e.g., Iran Health Insurance Organization and Social Security Organization) and social assistance schemes such as IKRC and the SWO. The purposive sampling method was applied, and the experts with relevant and valuable experiences were interviewed. The sampling continued until data saturation was achieved through a total of 33 semi-structured individual interviews. Each interview lasted for 20 to 126 min, and supplementary interviews were carried out when needed (as in two cases).

A reflexive semi-structured interview guide was developed for this study to conduct the interviews. The interview guide was developed based on the literature review, objectives of the study and the existing situation of user fee reduction in Iran’s healthcare financing system. It includes the open-ended questions which were asked from interviewees (Appendix). The validity of study questions was determined through initial interviews with three experts, and required changes were made in terms of concept, number, and sequence of questions.

The interview guide started from more general questions and gradually moved to specific questions. Based on the knowledge and experience of the participants, relevant and purposeful questions were asked to obtain more practical and detailed information. Efforts were made to narrow the topics down and bring in more probing questions about the user fee reduction for the poor.

The data collection was conducted in Persian, which is the official language in Iran. Participants of the study were recruited by face-to-face meetings. They were selected with a maximum variation sampling strategy based on their knowledge, experience, and professional position. The primary and open sampling procedures were used to recruit participants. The time and place of the interviews were determined based on the convenience of the participants. The first author conducted all the interviews. The main objective of the study was explained to the participants. Participants right to refuse to take part in the research at any time during the study was also stressed. Only two experts decline the invitation due to their busy schedules.

All interviews were recorded using an electronic device and transcribed word by word. The data were analyzed based on thematic analysis. Once each interview was done, its content was transcribed and stored on the computer. In the next stage, the texts were read and reviewed several times to acquire a command of the data. The data were then broken down into semantic units (codes) in the form of sentences and paragraphs associated with the original meaning. In the next step, the semantic units were read several times, and each was given a proper code. In each interview, the sub-themes were separated and merged, reductions were made, and the main themes were finally identified. To determine main themes and link them with abstract categories, the content of the final thematic was reviewed several times. Then, the emergent themes were discussed through peer debriefing until the research team reached a final consensus.

Two researchers independently coded transcripts. They identified the items relevant to each primary and compared the coding to reach a consensus on a coding scheme. The researchers then discussed, explored the relationship between the codes, and reached an agreement on the sub-themes within each theme. To improve the validity of the analysis and findings, the researchers were involved in organized discussions and sought feedback from some of the participants of the study.

Codes and themes were developed and reviewed by both authors to control the probable bias. Sufficient opportunities were provided to the participants to express their deep understandings of the context to control the potential sources of bias. The perspectives of colleagues were also used in coding and analyzing interview transcripts.

A detailed summary of each interview was used to extract the quotes, and open coding was done. The extracted codes were then categorized into three groups. The illustrative quotes that were explicitly distinctive of each code were presented. The selection of quotes for publication was discussed between authors. To give the reader the chance to assess alternative interpretations, results are reported with support from representative quotes. Thematic analysis enabled us to analyze data with reference to the main concepts/themes and provide supporting quotes related to these concepts.

The trustworthiness criteria were used to assure the quality of the results. The researchers spent sufficient time on data collection and retransmission to ensure the accuracy and robustness of the data. Before conducting the main study, several pilot interviews were carried out to improve the accuracy and credibility of the data collection. The transcribed content of the interviews was also given to some participants for respondent validation. To increase dependability, the researcher’s codified interview was re-examined by her colleague and the disagreement was resolved through consensus. Furthermore, diversity was taken into consideration in the participants’ organizations, provision of broad information coverage required at the highest possible levels, and increasing data transferability. The interviews were also directed for collecting the data with the researchers’ reflexivity. A triangulation approach, including interviews with key informants and comparing with other informative sources to find out the complementary of findings, was applied to increase data confirmability. Data were collected through semi-structured interviews and field notes to ensure methodological triangulation. The interviews were searched for negative or disconfirming opinions that do not fit the dominant pattern to improve trustworthiness. Not finding disconfirming results strengthened the confidence to generalize the findings. The maximum variation sampling technique was used to enhance the reflexivity and transferability of qualitative findings.

Ethical issues were considered in the present study. The participants were explained about the main research objective, and their written informed consent to participate in the study was obtained. They were informed that the interviews would be recorded and were also ensured that their names and information would remain confidential. The data were analyzed using the MAXQDA10 software.

### Findings

Table 1 reports characteristics of social health assistance programs for the poor in Iran. The most important issue about policies designed to protect the poor against user fee is policy coherence. The difference in access to financial support for user fee coverage and OOP between different groups of insured poor people has led to differences in access and utilization of health services among the poor. Thus, the first step is to integrate cohesive social assistance policies for disadvantaged groups in Iran.

**Table 1:** Characteristics of social health assistance programs for the poor in Iran: coverage population and benefits

Poor groups			Insurance fund	Premium	Cost-sharing support	Delivery system	Rationing mechanisms
Poor people on the social assistance scheme	Imam Khomeini Relief Committee	Women-headed households, families of needy prisoners, orphans living in urban areas	Other Sectors Fund	100% subsidized	Supplementary insurance of the relief committee	Family physician and referral system	–
	State Welfare Organization	Poor disabled, women-headed households, beggars, street children	Other Sectors Fund	100% subsidized	Only the disabled have a separate budget line for supplementary insurance in the welfare organization	–	–
Poor rural			Rural Insurance Fund	100% subsidized	Supplementary insurance of the relief committee plus paying less co-payment for visits and tests in rural health centers	Family physician and referral system	–
Marginalized people, people with informal jobs and insolvent			Universal Health Fund	100% subsidized	–	Family physician and referral system	Limited to public hospitals

The analysis of the interview transcripts resulted in the emergence of three themes and five sub-themes (see Table 2). Financial protection policies for the user fee at the point of service delivery can be divided into three main themes: 1) basic health social insurance instruments, 2) free health services to the poor outside of the health insurance system, and 3) complementary insurance mechanisms.

**Table 2:** Themes and sub-themes emerged from interview transcripts

Themes	Sub-themes (Number of participants mentioning the sub-theme)	Sample quotations
Basic health insurance instruments	User fee reduction mechanisms [25]	*“If I covered by the Relief Committee and spend on my health services up to a certain ceiling, it’s all the responsibility of the Committee or the health insurance organization to pay for the remaining. For example, if I filled the stop-loss ceiling by August, the insurance organization has to pay for it whenever I get sick. It’s not my share anymore. The stop-loss, for example, can be 15% of your annual income. We do not have such a thing, and that’s why OOP is constantly increasing.” P23*
	User fee exemption [17]	*“This is the principle. It is a mistake to receive money from the poor. If you have a referral system, there is no reason to pay because the system guides the patients. Because diseases are unpredictable, they are similar to accidents that can push the rich to poverty. Therefore, if there is an effective referral system, it should not be direct payment at all.” P2*
Free health services to the poor outside of the health insurance system	MOHME’s direct reimbursement to hospitals [23]	*“The MOHME can do this, and the poor can stay in hospitals for months, weeks, and days without any financial problem. For basic services, we should not receive user fee from the poor, and they should be covered using public resources.” P25*
	Social assistance organization’s reimbursement to the hospitals [16]	*“Mechanisms like this, for example, if poor patients went to a hospital and paid a user fee, they can come to us in the IKRC, we will reimburse the user fee to him/her. Pay the money, come and get it from us. If they could not pay, the Social Work Department in the hospital records the expenditures in the miscellaneous claims account. No need to receive money from the poor. “P23*
	Charities and NGO’s reimbursement to the hospitals [13]	*“Our assessments and the documents provided by the patient show that the person has incurred expenses. His illness is determined for us based on the prescription he receives or his medical certificate. As a medical charity, we cover as many patients as we can each year.” P23*
Complementary health insurance		

#### Basic health insurance instruments

##### User fee reduction mechanisms

Experts proposed various policy instruments to reduce the financial burden of user fee for the poor. Applying stop-loss and stepwise cost-sharing is an example of the policies.

"It becomes stepwise user fee. Many things can be done for the poor. The stepwise user fee is designed for this. Insurance organization can design stepwise user fee. It can say that for certain people with special diseases, I increase the coverage up to a given percentage. User fee can become close to zero for the very poor patients."P10

The stop-loss system can be designed in such a way that the share of the insurance organization is paid for the expenses under the ceiling, and from the specified ceiling onwards, all costs are paid by the insurance organization.

"If you do not define stop-loss in health insurance funds – in other words, you do not define the line for determining the out-of-pocket ceiling – vulnerable groups such as those covered by social assistance schemes will be severely pressured. If I covered by the Relief Committee and spend on my health services up to a certain ceiling, it’s all the responsibility of the Committee or the health insurance organization to pay for the remaining. For example, if I filled the stop-loss ceiling by August, the insurance organization has to pay for it whenever I get sick. It’s not my share anymore. The stop-loss, for example, can be 15% of your annual income. We do not have such a thing, and that’s why OOP is constantly increasing." P23

##### User fee exemption

Protecting the poor against catastrophic health payments by exempting patients from paying contributions increase their financial access to health services. Some participants emphasized the need for a user fee exemption policy for the poor, arguing that in addition to premiums, the user fee of the poor should be covered as they prevent them from using health services.

Treatment delay due to financial barriers among the poor leads to significant health and economic consequences to the society. Thus, user fee removal for the poor in the early stages of the disease diagnosis is more cost-effective for the government than spending a considerable amount of resources on the treatment of the final stage of the diseases and social assistance programs supporting households suffering from multidimensional poverty due to incurring catastrophic medical costs and loss of income because of illness.

"If patients are indigent, their user fees should be covered. If there are deprived people, all their needs should be met, and there should be no barriers for them to use healthcare services. This is because if low socioeconomic status patients are going to pay some out-of-pocket expenses, they may not realize that not using healthcare services will do serious damage to their health and future economic status and even to their next generation. I mean, if these patients are going to pay, they probably do not use the recommended treatments at all. Thus, it is better to cover these group of the population fully."P21

Some participants considered the exemption of the poor from user fee subject to a referral from general practitioners, including family physicians. The referral system ensures receiving healthcare services based on actual needs and, therefore, can control costs. In such a system, the poor can be exempted from user fee.

"This is the principle. It is a mistake to receive money from the poor. If you have a referral system, there is no reason to pay because the system guides the patients. Because diseases are unpredictable, they are similar to accidents that can push the rich to poverty. Therefore, if there is an effective referral system, it should not be direct payment at all." P2

However, some participants opposed the zero-user fee for the poor due to the concern about moral hazard (i.e., inappropriate overuse of services) among the insured. The concern with moral hazard was the main reason most experts favored a minimum user fee for the poor.

"You can reduce the user fee or remove it from a certain ceiling. These are several models, including a fixed deductible model. For example, patients do not pay after a hundred dollars. There is a payment ceiling because patients do not have money to pay. Paying a small amount of money makes them value the health services they receive.”P 29

The fee-for-service payment system provides incentives to induce demand for services deemed unnecessary. With the absence of user fee, providers are more likely to induce demand because their patients do not have financial worries about receiving services; thus, any policy to remove user fee should consider the supplier-induced demand that may arise from abolishing user fee in the healthcare system.

"You can provide some services such as inpatient services that are not subject to induced demand for free. Patients with special diseases such as dialysis patients who must do dialysis can access these services for free. In contrast, some services like paraclinical and rehabilitation cannot really be provided for free. This is because as long as there is a fee-for-service payment system in the country, you cannot provide these services for free due to the presence of supplier-induced demand. "P1

Exemption or reduction of user fee can be made through the issuance of a referral letter (or electronic communication) by the social assistance schemes (e.g., the IKRC and WO) that can be provided to healthcare providers (e.g., hospital). A major issue was how covered individuals apply to receive services. The referral system is a type of cost control mechanism and financial management to compensate for exemption costs, which seems necessary for the continuation of exemption schemes.

#### Free health services to the poor outside of the health insurance system

Financing free health services for the poor requires mechanisms to reimburse hospitals. The following three different sources were suggested.

##### MOHME’s direct reimbursement to hospitals

According to some experts, one of the inherent duties of the MOHME in Iran is to finance free healthcare services to the poor to avoid financial challenges in this group. The poor can be determined periodically based on income and employment status. Direct reimbursement by the government to hospitals is a practical solution to prevent some poor patients from not being able to discharge from hospitals due to inability to pay (no pay, no discharge is a common practice among some hospitals in Iran).

“I think the MOHME, as a service provider, should provide free services to the poor. The coverage can be provided for six months as the person may get a job later. The poor people identification code and their exemption time can be registered in an online system. They can be identified in all hospitals they referred to so that they receive health services for free. The MOHME can do this, and the poor can stay in hospitals for months, weeks, and days without any financial problem. For basic services, we should not receive user fee from the poor, and they should be covered using public resources.” P25

It should be such that all people covered by social assistance schemes should be treated free of charge. Physician visits in clinics and the use of medicines should be recorded and managed electronically. Hospitals should access the poor’ information and accept them.”P4.

##### Social assistance organization’s reimbursement to the hospitals

Another solution is not to receive user fee from the poor patients directly and record these expenses in the miscellaneous claim account of the defined institution to compensate the costs.

“Mechanisms like this, for example, if poor patients went to a hospital and paid a user fee, they can come to us in the IKRC, we will reimburse the user fee to him/her. Pay the money, come and get it from us. If they could not pay, the Social Work Department in the hospital records the expenditures in the miscellaneous claims account. No need to receive money from the poor. ”P23

##### Charities and NGO’s reimbursement to the hospitals

Another way to compensate the cost associated with the services received by the poor is sending the poor patients’ hospital bills to charitable organizations. This can be done if there is a defined mechanism for the reimbursement process. There should be responsible institutions for reimbursement to guarantee the sustainability of compensation of hospital revenue losses.

"Our assessments and the documents provided by the patient show that the person has incurred expenses. His illness is determined for us based on the prescription he receives or his medical certificate. As a medical charity, we cover as many patients as we can each year." P23

#### Complementary health insurance

The basic health insurance package should be provided equally to all people. The complementary insurance should cover user fee and the cost of services outside the basic package. The complementary health insurance is currently provided to the IKRC clients and in two provinces as a pilot for all individuals covered by the Rural Insurance Fund.

*"Who should pay the user fee? The SWO or IKRC? They should* integrate their financial supports *and work as a single complementary insurance organization. It is better to work together because one person may receive money from both." P13*

Indirect treatment costs, such as transportation costs, need to be funded by complementary insurance for identified poor.

“*This is called alternative insurance. It is neither complementary nor basic insurance. An alternative, a combination of complement and basic, insurance can be formed which can cover costs associated with accompanying persons and transportation to healthcare facilities.” P10.*

## Discussion

Similar to other health policies, the successful reduction of user fee for the poor depends on identifying and dealing with a wide range of different factors rooted in the contextual factors in the country. This issue is of paramount importance, particularly in developing countries that are faced with limited resources and different structural, institutional, and political conditions. This study aimed to explore the policy options for reducing user fee in the point of service among the poor. Results indicated that there are different ways to reduce user fee for the poor in Iran, which can be classified into three categories: basic health insurance instruments, free health services outside the health insurance system, and complementary health insurance.

### Basic health insurance mechanisms

According to the results, mechanisms defined to reduce costs in the health insurance systems can work to protect the poor. These mechanisms include a deductible, a fixed amount individuals pay each year before the insurance organization covers the rest. This type of payment should be set as a stepwise reduction in the price faced by the patient. For minor illnesses, the patients should pay the total cost, which is usually small. In case of serious illnesses, the patients should only pay the rate above the previous amount to reach the deductible, and then the insurance covers the remaining costs [28]. Another mechanism that can decrease the cost is stop-loss coverage, which provides adequate financial protection for patients after reaching a certain threshold [29]. In other words, after reaching a certain threshold of healthcare costs, the rest of health expenditures during a fixed period (one year) are covered by insurance organizations. This protects people from incurring further catastrophic and impoverishing healthcare expenditures. The poor should have a lower payment ceiling due to their low income [30]. Stepwise cost-sharing is another mechanism to protect the poor, which apply related cost-sharing rates of individuals to their ability to pay [31].

International experiences suggest that most countries reduce co-payment and other cost-sharing mechanisms for specific demographic groups such as low-income groups, the chronically ill, and children [32]. In Japan, people earning less than the national average and seniors (aged 70+), pay 10% co-insurance for medicine, while the rate for others is 30%. In addition, the monthly OOP limit is $1903 for high-income people and $449 for low-income people, and above these limits, only 1% user fee applies. Low-income people covered by the livelihood protection program are exempt from user fee. In terms of inpatient services, seniors earning a low or average income have 10% user fee while it is 30% for others. In the UK, 50% of people, including low-income people, children under 16, seniors (aged 60+), patients with cancer or chronic diseases, and people with high medicine prescriptions are exempted from the drug cost-sharing. In the US, low-income adults, pregnant women, seniors, children, and people with disabilities are covered by Medicaid insurance. These individuals are exempted from paying cost-sharing for inpatient and outpatient services and medications. In France, low-income people covered by the Public Health Insurance Act, children with disabilities and prisoners are exempted from paying user fee for inpatient services. In Germany, low-income groups do not pay for drug cost-sharing [33]. In Taiwan, low-income people, including people who earn less than 60% of the average individual consumption level and people with chronic illness, are exempted from cost-sharing. In Australia, the maximum amounts of OOP are set to $300 for low/middle-income people and $700 for others. This has led to a significant reduction in OOP [34].

### Free health services to the poor outside of the health insurance system

User fee exemption was one of the proposed policies by the participants to support the poor financially. User fee provides a small revenue for healthcare systems, and when its administrative costs are considered, the overall effect is negligible. As the benefits of user fee removal in terms of increasing service consumption surpass the cost of revenue loss, eliminating this source of income in the healthcare system is considered both equitable and efficient [35]. Most countries have implemented user fee exemption mechanisms. User fee in proportion to income or elimination of user fee for some services has been implemented in several countries to benefit the poor [36]. User fee waiver (a system that qualifies the poor for free or very low-cost services) and exemption (a system that provides certain services to patients for free or at a low price) are mechanisms designed to improve equity in access to health services in countries where patients pay the provider at the time of service [37]. Many patients may not use services due to inability to pay, or they may reduce consumption of basic necessities (e.g., food), use self-medication, visit local therapists, or borrow and sell their assets to cover catastrophic health costs associated with paying cost-sharing [38]. Evidence also suggest that user fee removal alone is not sufficient to remove barriers to access to services for the poor because indirect costs such as travel and informal payments also put financial pressure on them [39]. Therefore, complementary health insurance also required to remove barriers caused by indirect costs to use healthcare services by the poor.

### Complementary health insurance

Complementary coverage of treatment expenditures was one another policy proposed by the participants of this study. This policy has been implemented to reduce OOP for healthcare services in some countries. For example, as complementary to social health insurance program, Financial Health Assistance Program (a dual benefit package including subsidizing the poor to enroll in a social health insurance program and provide assistance cash to protect against catastrophic health expenditures) aims to protect the poor from financial risks associated with the diseases. Results suggested that the program is not an effective supplement to social insurance in terms of insurance registration and financial protection. As the program provides financial assistance to only 24% of the poor, there is a large gap between the cash benefit threshold and the average cost of services. It only covers the cost of a limited number of serious illnesses. This indicates a need to expand the cash assistance benefit package in China [40]. France’s free complementary insurance program has increased access to healthcare for seniors. People who had the most financial barriers to access services benefited the most from the program due to the lack of complementary coverage. The total cost of implementing the program was lower than expected. Doctors’ refusal to provide services to insured persons covered by the free program and the difference in the quality of care provided to them have been the two challenges of the program [41].

#### Interpretation

According to Article 27 of Iran’s Fifth Development Plan Law, the Government is allowed to establish a comprehensive multi-tier social security system, the first layer of which is social assistance, including support and empowerment services. Based on this Article, all the poor covered by the protection system are exempted from insurance premiums and their user fee for the services in the public sector are covered by the relevant support institutions. If the poor patients use healthcare services from non-governmental and private health sectors, the patients are responsible for paying the approved cost-sharing and the difference between the private and public health tariffs [42]. Although the system has not been fully implemented yet, the limited support to cover the user fee for the poor if they use their needed healthcare services from non-governmental and private health sectors increases the risk of incurring catastrophic expenditures. Also, some poor individuals covered by specific organizations (e.g., Health Insurance Organization) can only receive healthcare services from public hospitals, and they must pay the entire cost of services if they use private hospitals. A recent study suggested that these individuals have the highest rate of catastrophic health expenditures among those who used healthcare services from the private sector in Iran [43].

Evidence shows that drug and pharmaceutical spending accounts for 43% of total OOP in Iran. Outpatient services (24%) and inpatient services (14%) also accounts for a significant share of OOP in Iran [44]. More than 63% of outpatient services in Iran are provided by the private sector [45]. There is no user fee reduction for service provided by the private sector [46], which has resulted in inefficiency in Iran’s healthcare system as the poor instead of pursuing services in the outpatient departments, seek inpatient services in public hospitals. This is because the coverage for outpatient services are not as comprehensive as inpatient services for the poor in Iran. This, in turn, has led to a reduction in OOP for the outpatient sector over time and an increase in OOP for inpatient services in Iran [41].

International development agencies, including United Nations organizations (e.g., the Word Bank), have adopted social protection policies within the health sector, focusing mainly on user fee removal and subsidized insurance schemes. As these policies rarely could address a primary concern of health insecurity (inability to provide adequate healthcare services today and the risk of not being able to provide in the future) in low/middle-income countries, there is a need to integrate social protection programs to address poverty brought by OOP for healthcare services [47]. In other words, it is necessary to combine these measures with the social protection system by breaking the wall between the social and health sector. The International Labor Organization instruments stipulate legal entitlements to healthcare “without hardship”. OOP payments should not be a primary source for financing healthcare systems. The rules about cost-sharing must be designed to alleviate financial hardship, with limited co-payments [48].

Health systems should have mechanisms to reduce user fee for the poor to ensure adequate access to health services. They should ensure that no one postpones treatments or withdraw services because of financial inability. Iran’s health system does not have a specific user fee protection policy for the poor. There is an equal user fee system for all citizens, except some programs that reduce user fees for special diseases regardless of patients’ income level. Specifically, the poor in Iran face higher catastrophic healthcare payments when they use healthcare services from the private sector. The recent annual budget law has obliged the health insurance organizations to reduce user fee for the poor in the private sector if they receive their needed services through a referral system. However, this has not been implemented yet.

Previous literature in Iran highlighted the financial gap in the access to health services for the poor and the necessity to design specific protective measures for them [49–51]. This study attempted to fill this research gap through an in-depth analysis of health policymakers’ opinions about potential policies to improve access to health services among the poor. The findings provided preliminary but important information about Iranian professionals’ viewpoints on designing policies to improve financial protection to achieve universal access to health services.

### Strengths and limitations

The main strength of this study is that the researchers interviewed a wide variation of policymakers to gather all perspectives about user fee removal to enhance the trustworthiness of findings. Like other qualitative studies, this study is subject to some limitations. First, the findings should be interpreted with caution as it is not conclusive which policies mentioned by the participates may work in the real world. Second, the participants mentioned some opposite viewpoints about the options of the user fee reduction for the poor. It is not clear which participant provided a more accurate interpretation of the reality about user fee removal policies. Third, although collecting information from a diverse group of policymakers and analyzing data by peer debriefing enhanced the trustworthiness of the study findings, the interpretations may persist subjectively.

## Conclusion

Reducing user fee among the poor through mechanisms such as deductible, cap, stop-loss, variable user fee, and sliding fee scale can potentially reduce some of the adverse effects of under-utilization of healthcare among the poor in Iran. However, a new user fee system should be designed to cover the poor in a comprehensive protection package to reduce both OOP at the time of service delivery and indirect costs of receiving service. This study lays the ground for further efforts to study and implement financial protection policies in Iran’s healthcare system. The findings can be used in countries with similar social and economic contexts to design policies aimed at improving healthcare care use among the poor. Future work should explore more influencing factors on implementing user fee removal policy for the poor. Furthermore, future studies that aim to investigate barriers to using healthcare services with no user fee can provide valuable information for policymakers to develop effective healthcare programs for the poor.

## Data Availability

The data used for the study are available upon reasonable request from the corresponding author.

## Appendix

### Interview Guide

Date and venue:

Name and position of interviewee:

1. What is the reason to consider removing the user fee for the poor? What is your opinion about the policy?
2. Which options for the user fee removal have been discussed? What are your thoughts on different policy options?
3. Regarding the removal of user fee policy, to what extent do you think Iran’s health system has the capacity to create and implement the policy? Which factors should be considered before the implementation of the policy?
4. What will be the effects of the policy (positive and negative) on service providers, the health system and poor patients?
5. What are the effects of the policy on the financing of public hospitals?
6. Which parties/stakeholders are relevant to user fee removal policy? What are the views of policymakers and stakeholders (e.g., insurance organizations, medical council organization, Ministry of Health and Medical Education, and people) about the implementation of the policy?
7. How does your institution organize pro-poor resource allocation, decision making and budgeting? and what are the challenges?
8. How do you see the role of charities?
9. Do you have any other comments/thoughts about the policy?

**Note:** The questions were adjusted based on each interviewee’s position in the health system.

## References

[1] Kutzin J (2000). Towards universal health care coverage: goal-oriented framework for policy analysis.

[2] Bennett S, Gilson L (2001). Health financing: designing and implementing pro-poor policies.

[3] Holst J (2010). Patient cost sharing: reforms without evidence.

[4] Pearson M (2004). Issues paper: the case for abolition of user fees for primary health services.

[5] Robertson CT (2014). Scaling cost-sharing to wages: how employers can reduce health spending and provide greater economic security. Yale J Health Pol’y L Ethics.

[6] Remler DK, Greene J (2009). Cost-sharing: a blunt instrument. Annu Rev Public Health.

[7] Chalkley M, Robinson R (1997). Theory and evidence on cost sharing in health care: an economic perspective.

[8] Honda A (2006). User fees policy and equitable access to health care services in low-and middle-income countries-with the case of Madagascar.

[9] Saloner B, Sabik L, Sommers BD (2014). Pinching the poor? Medicaid cost sharing under the ACA. Obstet Gynecol Survey.

[10] Nabyonga J, Desmet M, Karamagi H, Kadama PY, Omaswa FG, Walker O (2005). Abolition of cost-sharing is pro-poor: evidence from Uganda. Health Policy Plann.

[11] Taxing the ill (2017). How user fees are blocking Universal Health coverage.

[12] Witter S (2005). An unnecessary evil? User fees for healthcare in low-income countries.

[13] Qin VM, Hone T, Millett C, Moreno-Serra R, McPake B, Atun R (2019). The impact of user charges on health outcomes in low-income and middle-income countries: a systematic review. BMJ Glob Health.

[14] Ponsar F, Tayler-Smith K, Philips M, Gerard S, Van Herp M, Reid T (2011). No cash, no care: how user fees endanger health—lessons learnt regarding financial barriers to healthcare services in Burundi, Sierra Leone, Democratic Republic of Congo, Chad, Haiti and Mali. Int Health.

[15] Lépine A, Lagarde M, Le Nestour A (2018). How effective and fair is user fee removal? Evidence from Zambia using a pooled synthetic control. Health Econ.

[16] Lagarde M, Palmer N. The impact of user fees on health service utilization in low-and middle-income countries: how strong is the evidence? Bull World Health Organ. 2008;86(11):839–48. 10.2471/BLT.07.049197 PMC2649541 19030689

[17] Worldbank (2018). Poverty headcount ratio at $5.50 a day (2011 PPP) (% of population) - Iran, Islamic Rep.

[18] Mobaraki H, Rezapor A, Rahiminia R, Asadi H, Ghavamiazad Z, Jooyani Y (2018). Catastrophic Health expenditure and its determinants in older adults in Tehran, Iran. Caspian J Health Res.

[19] Rezaei S, Hajizadeh M (2019). Measuring and decomposing socioeconomic inequality in catastrophic healthcare expenditures in Iran. J Prev Med Public Health.

[20] Etemadi M, Tadayon MM (2019). Health Financial Protection for the Poor in the times of Economic Crises: Resistance Economy Policies. Iranian J Culture Health Promot.

[21] Etemadi M, Ashtarian K, Gorji HA, Kangarani HM (2019). Which groups of the poor are supported more by the law? Pro-poor health policy network in Iran. Int J Health Plann Manage.

[22] Etemadi M, Gorji HA, Kangarani HM, Ashtarian K (2017). Power structure among the actors of financial support to the poor to access health services: social network analysis approach. Soc Sci Med.

[23] Anjomshoa M, Akbari Sari A, Takian A (2021). Assessing progress in the national health financing system towards universal health coverage in Iran: a mixed-method study protocol. Health Res Policy Syst.

[24] Etemadi M (2018). Developing a financial support policy model for access of the poor to health services in Iran.

[25] Rezaei S, Woldemichael A, Ebrahimi M, Ahmadi S (2020). Trend and status of out-of-pocket payments for healthcare in Iran: equity and catastrophic effect. J Egyptian Public Health Assoc.

[26] Woldemichael A, Rezaei S, Kazemi Karyani A, Ebrahimi M, Soltani S, Aghaei A (2021). The impact of out-of pocket payments of households for dental healthcare services on catastrophic healthcare expenditure in Iran. BMC Public Health.

[27] Aryankhesal A, Etemadi M, Agharahimi Z, Rostami E, Mohseni M, Musavi Z (2016). Analysis of social functions in Iran’s public hospitals: pattern of offering discounts to poor patients. Int J Human Rights Healthcare.

[28] Phelps E, Health Economics C (1997). Tehran: new economics.

[29] Culyer AJ (2014). Encyclopedia of health economics: Newnes.

[30] Drèze JH, Schokkaert E (2013). Arrow’s theorem of the deductible: moral hazard and stop-loss in health insurance. J Risk Uncertain.

[31] Hall MA (2011). The mission of safety net organizations following national insurance reform. J Gen Intern Med.

[32] Paris V (2014). Health benefit plans in OECD Countries. LAC webinar.

[33] Hossein Z, Gerard A (2013). Trends in cost sharing among selected high income countries—2000–2010. Health Policy.

[34] Qingyue M, Liying J, Beibei Y (2011). Cost-sharing mechanisms in health insurance schemes: a systematic review. The Alliance for Health Policy Systems Research, WHO.

[35] Yates R (2006). International experiences in removing user fees for Health services–implications for Mozambique.

[36] Ashford LS, Gwatkin DR, Yazbeck AS (2006). Designing health and population programs to reach the poor.

[37] Bitrán R, Giedion U. Waivers and exemptions for health services in developing countries. Washington: World Bank; 2002.

[38] James C, Hanson K, McPake B, Balabanova D, Gwatkin D, Hopwood I (2005). To retain or remove user fees? Reflections on the current debate. Appl Health Econom Health Policy.

[39] Kruk ME, Mbaruku G, Rockers PC, Galea S (2008). User fee exemptions are not enough: out-of-pocket payments for 'free' delivery services in rural Tanzania. Trop Med Int Health.

[40] Liu K, Yang J, Lu C (2017). Is the medical financial assistance program an effective supplement to social health insurance for low-income households in China? A cross-sectional study. Int J Equity Health.

[41] Grignon M, Perronnin M, Lavis JN (2008). Does free complementary health insurance help the poor to access health care? Evidence from France. Health Econ.

[42] Gorjipoor E (2016). Policy documents on multi-layer social security system Tehran: social security organization research institute.

[43] Etemadi M, Shiri M, Rostami E, Mohseni M, Seyedi M (2019). Financial burden imposed on the insured patients for private treatment: Evidence from a state of Iran. J Educ Health Promot.

[44] Hsu J, Majdzadeh R, Harichi I, Soucat A. Health system transformation in the Islamic Republic of Iran: an assessment of key health financing and governance issues. Health system transformation in the Islamic Republic of Iran: an assessment of key health financing and governance issues. Washington: World Health Organization; 2020.

[45] NekoeiMoghadam M, Amiresmaili M, Izadi A (2016). The competitive analysis of the private hospital industry using Porter's competitive diamond model: a case study in Kerman 2014. J Qual Res Health Sci.

[46] Arani AA, Atashbar T, Antoun J, Bossert T (2018). Iran’s health reform plan: measuring changes in equity indices. Iran J Public Health.

[47] Gama E (2016). Health insecurity and social protection: pathways, gaps, and their implications on health outcomes and poverty. Int J Health Policy Manag.

[48] ILO (2021). World Social Protection Report 2020–22: Social protection at the crossroads – in pursuit of a better future.

[49] Etemadi M, Abolghasem GH (2019). Trade-off between efficiency and equity on the rationing in Health insurance system: the burden on the poor. Evid Based Health Policy Manage Econom.

[50] Abdi Z, Hsu J, Ahmadnezhad E, Majdzadeh R, Harirchi I (2020). An analysis of financial protection before and after the Iranian Health transformation plan. East Mediterr Health J.

[51] Vahedi S, Yazdi-Feyzabadi V, Amini-Rarani M, Mohammadbeigi A, Khosravi A, Rezapour A (2020). Tracking socio-economic inequalities in healthcare utilization in Iran: A repeated cross-sectional analysis. BMC Public Health.

